# Serum 25(OH) Vitamin D Levels in Pregnant Women with Coronavirus Disease 2019 (COVID-19): A Case-Control Study

**DOI:** 10.3390/ijerph19073965

**Published:** 2022-03-26

**Authors:** Nazaret Ferrer-Sánchez, Marina Díaz-Goicoechea, Victoria Mayoral-Cesar, Silvia García-Solbas, Bruno José Nievas-Soriano, Tesifón Parrón-Carreño, Ana María Fernández-Alonso

**Affiliations:** 1 Obstetrics and Gynaecology Unit, Torrecárdenas Hospital, 04009 Almería, Spain; marinadiazgoicoechea@gmail.com (M.D.-G.); mayoral.87@hotmail.com (V.M.-C.); anafernandez.alonso@gmail.com (A.M.F.-A.); 2 Obstetrics and Gynaecology Unit, Vithas Virgen del Mar Hospital, 04120 Almería, Spain; silviagarsol@gmail.com; 3 Department of Nursing, Physiotherapy and Medicine, University of Almería, 04120 Almería, Spain; tpc468@ual.es

**Keywords:** vitamin D, COVID-19, coronavirus, pregnant women

## Abstract

The physiological changes during pregnancy may increase the risk of complications in pregnant women with coronavirus disease 2019 (COVID-19). Vitamin D is a fat-soluble secosteroid hormone and its role in immunity is appears to be of particular importance in this recent pandemic. Nevertheless, there is little research about the role of vitamin D levels regarding COVID-19 in pregnant women to date. This study aimed to establish a relationship between serum 25-hydroxyvitamin D (25(OH)D) levels in pregnant women and COVID-19. A comparative case-control study was performed with a study population of 256 pregnant women (82 pregnant women with infection and 174 women in control group). Serum 25(OH)D levels were significantly lower in pregnant women with COVID-19 infection than in those without infection. In addition, 89% of COVID-19-positive pregnant women had 25(OH)D deficiency, while in the control group the percentage was 75.30%, finding statistically significant differences (ORa = 2.68; 95% CI 1.19–6.06; *p* = 0.01). Our results find a relationship between vitamin D deficiency in pregnant women and COVID-19 infection. This finding could be relevant for actual clinical practice. Thus, more research is needed in this field.

## 1. Introduction

Severe acute respiratory syndrome coronavirus 2 (SARS-CoV-2) emerged in Wuhan, China, and it spread rapidly throughout the world. The World Health Organization (WHO) declared a global health emergency on 31 January 2020, and subsequently, a pandemic on 11 March 2020. There are more than 400 million confirmed cases of people infected with this coronavirus, including nearly 6 million deaths worldwide at the time of writing [1]. Symptoms of SARS-CoV-2 infection in pregnant women are similar to those in the general population. However, pregnant women, when they present clinical symptoms, are more likely to require treatment in the intensive care unit (ICU) when compared to non-pregnant ones [2]. Some authors state that this is due to the different physiological changes during gestation, as pregnant women may have an increased risk of complications [3,4,5].

Vitamin D is a fat-soluble secosteroid hormone [6]. The classic functions that have been attributed to vitamin D are related to calcium and phosphate homeostasis, acting in the intestines, kidneys, and bones [7]. However, in recent years, other functions of vitamin D have been described, for example, its important immune function [8,9]. In this sense, vitamin D deficiency has been associated with an increased risk of developing autoimmune diseases [10] and respiratory infections [11]. Furthermore, vitamin D deficiency has been linked with adverse effects on pregnancy and offspring such as placental insufficiency [12,13], preterm delivery [14], gestational diabetes [15], low birth weight, and being small for gestational age [16].

As the outbreak of coronavirus disease 2019 (COVID-19) develops, the prevention and management of COVID-19 among pregnant women and possible maternal complications induced by the infection have become a major concern. Nevertheless, there is little research about the role of vitamin D levels regarding the COVID-19 in pregnant women. Therefore, our objective was to determine the relationship between serum 25-hydroxyvitamin D (25(OH)D) levels, specifically decreased levels, in pregnant women with COVID-19 infection and the association with the severity of the disease.

## 2. Materials and Methods

### 2.1. Study Design

An observational comparative case-control study was performed between singleton gestations with COVID-19 infection versus uninfected pregnant women who completed delivery in the same period.

This study was carried out in the Obstetrics and Gyneacology Department of Torrecardenas Hospital in the period between November 2020 and September 2021, with a subsequent 4-week follow-up of the patients included in the study. Torrecardenas Hospital is the reference hospital of the province of Almeria, Spain, with 727,945 inhabitants, and attends about 3000 deliveries per year.

### 2.2. Study Population

The study population consisted of singleton pregnant women who were admitted for ongoing labour or any other reason but who terminated their pregnancy by delivery or caesarean section during their admission.

Two groups were established: case group or pregnant women with COVID-19 infection and control group or pregnant women without infection. Inclusion criteria for the case group were: pregnant women who tested positive for SARS-CoV-2 using real-time quantitative reverse transcription (qRT-PCR) from nasopharyngeal and oropharyngeal samples, with single pregnancy and who had pregnancies with date of last known menstrual period and normal prenatal controls normal up to the time of diagnosis. Inclusion criteria for the control group were: pregnant women with negative qRT-PCR who completed delivery in the same period as the case group, with single pregnancy, and who had pregnancies with a date of last known menstrual period, and normal prenatal controls up to the time of diagnosis. Two control subjects were matched prospectively to each case and deliveries were immediately after the delivery of pregnant women with COVID-19 infection. Exclusion criteria for both groups were: pregnant women under 18 years old, who did not give informed consent at the time of inclusion in the study, who had multiple gestation, and who took extra vitamin D supplements during pregnancy.

### 2.3. Sample Size

**Scheme 1:**
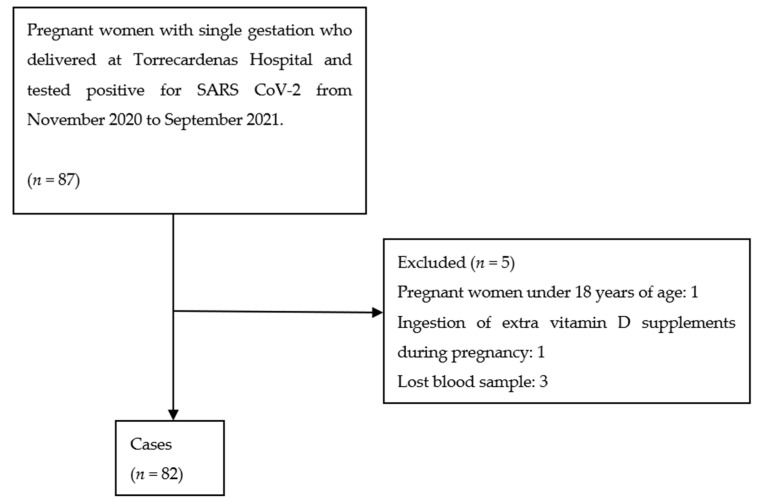
Case selection process.

The 2:1 ratio was based on a priori power calculations. It was necessary to recruit a minimum of 31 patients in the case group and 62 in the control group to achieve a power of 80% to detect differences in the contrast of the null hypothesis (Ho), taking into account that the significance level was 5% and assuming that the proportion in the control group was 5% and that the expected loss percentage was 10%. The case group included 87 pregnant women, and the control group 174 pregnant women. However, after study exclusions (Scheme 1), the final case group included 82 pregnant women.

### 2.4. Study Variables

The following variables were collected: maternal age; level of education; race (the race was included because skin colour influences vitamin D levels [17,18]); tobacco consumption; pre-pregnancy body mass index (BMI, calculated as weight in kilograms divided by the square of height in meters), pre-pregnancy obesity (pre-pregnancy BMI ≥ 30 kg/m^2^ was defined as obese); parity; chronic hypertension; pregestational diabetes; asthma; gestational hypertension; preeclampsia; gestational diabetes; gestational hypothyroidism; gestational week at the delivery; preterm birth; cause of preterm birth; labour beginning; labour ending; neonatal outcomes; and the following laboratory parameters at the time of delivery: 25(OH)D (ng/mL), haemoglobin, haematocrit, leukocytes, lymphocytes, platelets, prothrombin time, and fibrinogen. Variables of symptoms, type of symptoms, hospital admission criteria, admission criteria to the ICU, and severity were also collected by the researchers in women with COVID-19 infection. These pregnant women were classified as mild, moderate, severe, or critical according to the WHO Living guidance for clinical management of COVID-19 [19].

### 2.5. Data Collection

Pregnant women who met the study inclusion criteria, after obtaining informed consent, responded to an interview where some of the variables studied were collected. Subsequently, a blood sample was extracted at delivery, and medical records were reviewed. Serum 25(OH)D levels were measured on a Roche Modular E 170 analyser (Roche Diagnostics, West Sussex, England). Results are expressed in ng/mL (1 ng/mL = 2.496 nmol/L). This method has been validated against high-performance liquid chromatography (HPLC) and radioimmunoassay methodology and can detect 25(OH)D levels in the range of 4 to 96 ng/mL [20]. Levels of 25(OH)D were classified following the Spanish Society of Endocrinology and Nutrition (SEEN) as sufficient (≥30 ng/mL), insufficient (20–29 ng/mL), deficient (10–19 ng/mL), and a last classification was added called severe deficiency (<10 ng/mL). This variable was also grouped into a new variable called 25(OH)D deficiency, showed as a dichotomous qualitative variable: Yes/No. Vitamin D deficiency was defined when values were below 20 ng/mL, according to the SEEN criteria.

### 2.6. Ethical Implications

This study was approved by the Ethics Committee of the Torrecárdenas University Hospital on 27 January 2021, with protocol number 2607-143/2020. Ethical principles were taken into account and the confidentiality of the participants was respected, in compliance with the Declaration of Helsinki of the World Medical Association (WMA), which sets out the ethical principles for medical research on human beings.

Pregnant women who gave birth were informed about the observational nature of the study and asked to participate after giving written consent. The information was treated confidentially and anonymously.

### 2.7. Statistical Analysis

Statistical analysis was performed with SPSS version 25.0 software package (SPSS, Chicago, IL, USA). A descriptive analysis of the variables was carried out, calculating frequencies and percentages for those variables that were qualitative and measures of central tendency and dispersion for the quantitative variables. Prior to hypothesis contrast between quantitative and qualitative variables, a Kolmogorov–Smirnov test was performed to verify if they followed a normal distribution. According to this, nonparametric data were analysed with the Mann–Whitney U test (2 independent samples). The Pearson-X^2^ test was used to compare percentages, with Yates’ correction factor and Fisher’s exact test if necessary.

Finally, a regression study was carried out to complete the results. A binary logistic regression analysis to adjust for possible confounding factors was performed. The dependent variable used was COVID-19 infection and independent variables considered for the analysis were vitamin D deficiency, maternal age, education, and race. We divided the race into Caucasian and Arab women since they accounted for the majority of the sample, discarding the rest to avoid possible biases.

A value of *p* < 0.05 was considered statistically significant in all the hypothesis contrast tests.

## 3. Results

A total of 256 women participated in this study. Case group consisted of 82 (32%) women, while 174 (68%) women had no positive qRT-PCR for SARS-CoV-2.

Bivariant analyses showed that statistically significant differences were observed when comparing both groups regarding their level of education. Sixty-five (37.60%) pregnant women in the control group had a university education, compared to seven (8.60%) in the COVID-19 group (*p* < 0.001). The rest of the educational levels were higher in pregnant women with COVID-19 infection. The percentage of Caucasian women was significantly higher in the control group versus the COVID-19 group. However, the percentage of Arab, Hispanic, and Black races was higher in women with COVID-19 infection compared to women in the control group (*p* = 0.001). Preterm deliveries were more frequent in the case group women (19.50%) compared to the control group (5.20%), this difference being statistically significant (*p* < 0.001). The newborns of the case group´s mothers had a significantly lower weight than of those in the control group (*p* < 0.001), and they had significantly more foetal growth restriction (*p* = 0.02). In addition, there were significantly more newborns with Apgar score < 7 at 5 min in mothers with COVID-19 infection (4.9%) than in the control group (0.00%) (*p* = 0.002).

We found no statistically significant differences in the rest of the characteristics of patients and delivery (Table 1) between both groups, nor in the laboratory parameters: haemoglobin, haematocrit, leukocytes, lymphocytes, platelets, prothrombin time, and fibrinogen.

Serum 25(OH)D levels of the participant of the study are shown in Table 2. We found low serum 25(OH)D levels in both groups, but they were significantly higher in the control group, with a median of 13.80 [11.45] ng/mL, compared to the COVID-19 group, with a median of 10.15 [9.45] ng/mL (*p* = 0.005). Furthermore, statistically significant differences were found in vitamin D deficiency status. Vitamin D sufficiency, insufficiency, and deficiency were more frequent in the control group women, while severe deficiency was higher in women with COVID-19 infection (*p* = 0.004). Seventy-three (89%) pregnant women in the COVID-19 group had vitamin D deficiency (severe or not), while 131 (75.30%) women in the control group were deficient (*p* = 0.01, ORc = 2.66, 95% CI: 1.22–5.77). After adjusting for confounding factors, vitamin D deficiency was still statistically significantly different between both groups (*p* = 0.01, ORa = 2.68, 95% CI: 1.19–6.06) (Table 3). A binary logistic regression analysis, using COVID-19 as the dependent variable, and age, obesity, pre-pregnancy weight, weight in the third trimester of pregnancy, and type of delivery as independent variables, was performed. The analysis showed that the independent variables were not influential.

In the case group, 7 (8.50%) women had a severe, moderate, or critical infection, while 75 (91.50%) women had a mild infection. Four (4.90%) patients required ICU admission and 25 (30.50%) women had symptoms. Symptoms were sore throat (12%), fever (40%), anosmia (20%), ageusia (8%), cough (64%), respiratory distress (28%), and headache (28%). Thirty-six percent of women with COVID-19 infection also had pneumonia. The main reason for hospital admission was labour or obstetric reasons in 76 (92.7%) of the women, while only 6 (7.3%) of the pregnant women were admitted due to COVID-19 infection. Nasopharyngeal qRT-PCR of the newborns was positive in 2 cases.

Regarding vitamin D deficiency, an analysis of pregnant women with COVID-19 infection who had a mild infection versus moderate, severe, or critical infection was performed. Although serum 25(OH)D levels were higher in pregnant women with a mild infection, no statistically significant differences were found (*p* = 0.25). All patients with moderate, severe, or critical infection had vitamin D deficiency (*p* = 0.19) (Table 4). There were also no statistically significant differences in vitamin D deficiency in women who were admitted to the ICU versus those who were not (Table 5).

## 4. Discussion

The main aim of this research was to analyse the relationship between decreased serum 25(OH)D levels in pregnant women with COVID-19 infection. We also sought to assess its association with the severity of infection.

The results of our study show a relationship between vitamin D levels and COVID-19 infection in pregnant women. Although levels were low in both groups, vitamin D levels were significantly lower in those women who had the infection. In addition, 89% of infected pregnant women had 25(OH)D deficiency (<20 ng/mL) while 75.30% of negative pregnant women had 25(OH)D deficiency, which was statistically significant. Adjusting for possible confounding factors, we found that pregnant women with 25(OH)D deficiency were 2.68 times more likely to have COVID-19 infection.

Investigations such as this one assessing 25(OH)D levels in pregnant women with COVID-19 infection are scarce to date. Vitamin D levels were also significantly lower in COVID-19-positive compared to -negative pregnant women, in a case-control study conducted in Ankara. These vitamin D levels were also statistically significantly lower in those women with moderate or severe COVID-19 infection compared to those with milder infection [21]. However, another cohort study in pregnant women found no statistically significant differences in vitamin D deficiency between pregnant women with COVID-19 infection and control group, nor the severity of infection [22]. Mean 25(OH)D levels were significantly lower than the cut-off values (30 ng/mL was taken as the cut-off value) in a published study of 44 pregnant women with COVID-19 infection. However, this study has limitations as it does not compare the results with uninfected pregnant women (controls), so it is not possible to establish causality [23].

Concerning the non-pregnant population, some authors are trying to find a possible relationship between vitamin D and COVID-19 infection. A study in Israel involving 7807 individuals (10.02% positive and 89.98% negative qRT-PCR for SARS-CoV-2) found that serum 25(OH)D levels were lower in positive than negative individuals. A significant and independent association was demonstrated between decreased 25(OH)D levels and increased likelihood of COVID-19 infection. These 25(OH)D levels were also associated as a risk factor for SARS-CoV-2 hospitalization [24]. Another study in Florida also showed an association between vitamin D deficiency and the risk of developing COVID-19 infection, even after adjusting for confounding factors [25].

Moreover, decreased 25(OH)D levels have not only been associated with an increased likelihood of SARS-CoV-2 infection but have also been associated with disease severity and mortality in non-pregnant population [26,27,28]. Our study also assessed the vitamin D levels regarding the severity of the infection and the need for admission of the pregnant woman to the ICU. Although in the present study we found that pregnant women with moderate, severe, or critical infection had lower 25(OH)D levels compared to women with mild infection, this association was not significant. The same was true for women who required admission to the ICU. This may be because of the 82 women with COVID-19 infection, only seven women had moderate, severe, or critical infection, and four required ICU admission; these results may not be representative of trends in the general population.

Our results could be explained by the important role of vitamin D in the immune system. Its relationship with the prevention of respiratory infections and local inflammation at the pulmonary level has been established [11,29,30]. However, this alone does not explain its possible relationship to COVID-19 infection. SARS-CoV-2 decreases angiotensin 2-converting enzymes (ACE-2) and increases toxicity due to angiotensin II accumulation which, in turn, influences respiratory syndrome and myocarditis [31]. Vitamin D regulates the renin–angiotensin–aldosterone system (RAAS), decreasing renin and ACE expression and promoting ACE-2 expression, which may explain the possible protective role against acute lung injury [32,33]. Vitamin D may also influence the cytokine storm through its anti-inflammatory role in the immune system by decreasing the production of proinflammatory cytokines and increasing anti-inflammatory cytokines [31].

Furthermore, other studies show a relationship between vitamin D and some immune system factors. One study suggested that complement induces in T Helper 1 cells (Th1) the expression of vitamin D receptors and the CYP27B1 enzyme (which activates vitamin D), causing these cells to respond to vitamin D to suppress interferon-γ (IFN-γ) and increase interleukin-10 (IL-10). This could stop or modulate the hyperinflammatory response in patients with COVID-19 [34]. A link has also been described between vitamin D deficiency and decreased numbers of natural killer (NK) cells, preventing the cell barrier from forming during early viral infections [35].

Our research has some limitations. One of the limitations of this study is that only pregnant women with COVID-19 infection at the time of delivery were analysed and we did not analyse the possible impact of the infection at another time during pregnancy. COVID-19 vaccination was not yet recommended for pregnant women at the time of our study, so none of the women included in the study had been vaccinated. As the association between 25(OH)D levels and severity of infection could be affected by this intervening variable, this aspect could be considered for future research. In our study, no information about the diet and exposure to the sun was collected. As these variables could affect vitamin D status, they could be considered when performing similar research. Other limitations were the small sample and the low number of COVID-19-positive pregnant women with severe infection. However, despite the small sample, we completed the necessary number of cases required by the sample size to achieve a power of 80% to detect differences in contrast to the null hypothesis. Moreover, our study offers interesting results that may have relevance for clinical practice.

## 5. Conclusions

Our results show a relationship between vitamin D deficiency in pregnant women and COVID-19 infection. However, it is necessary to confirm with other studies whether decreased vitamin D levels may be a risk factor for developing COVID-19 infection upon coronavirus exposure. These findings can be relevant for actual clinical practice. Therefore, more research is needed in this important field.

## Figures and Tables

**Table 1:** Characteristics of patients and delivery.

Characteristics	COVID-19 Group (*n* = 82) [Interquartile Range] or *n* (%)	CONTROL Group (*n* = 174) [Interquartile Range] or *n* (%)	*p*
Age (years)	31 [8]	32 [9]	0.83 *
Level of education ^†^			
Non studies	13 (16)	18 (10.40)	
Primary studies	25 (30.90)	33 (19.10)	
Secundary studies	36 (44.40)	57 (32.90)	<0.001 ^‡^
University studies	7 (8.60)	65 (37.60)	
Race			
Arabic race	29 (35.40)	39 (22.40)	
Black race	4 (4.90)	3 (1.70)	
Hispanic race	5 (6.10)	3 (1.70)	0.001 ^§^
Caucasian race	44 (53.70)	129 (74.10)	
Tobacco consumption			
Yes	4 (4.90)	17 (9.80)	0.16 ^||^
Pre-pregnancy BMI (kg/m^2^)	25.44 [7.39]	24.26 [6.37]	0.18 *
Pre-pregnancy obesity			
Yes	18 (22.20)	27 (15.60)	0.19 ^‡^
Parity Primiparous Multiparous	28 (34.10) 54 (65.90)	75 (43.10) 99 (56.90)	0.17 ^‡^
Chronic hypertension			
Yes	1 (1.20)	0 (0.00)	0.13 ^||^
Pregestational diabetes			
Yes	0 (0.00)	0 (0.00)	NA
Asthma			
Yes	2 (2.40)	9 (5.20)	0.29 ^||^
Gestational hypertension			
Yes	0 (0.00)	2 (1.10)	0.21 ^||^
Preeclampsia			
Yes	3 (3.70)	1 (0.60)	0.07 ^||^
Gestational diabetes			
Yes	12 (14.60)	17 (9.80)	0.25 ^‡^
Gestational hypothyroidism			
Yes	5 (6.10)	23 (13.20)	0.08 ^‡^
Gestational week	40 [3]	40 [2]	0.006 *
Preterm birth			
Yes	16 (19.50)	9 (5.20)	<0.001 ^‡^
Cause of preterm birth Spontaneous Maternal indication Fetal indication	7 (43.80) 5 (31.30) 4 (25)	3 (33.30) 3 (33.30) 3 (33.30)	0.58 ^§^
Labour beginning Spontaneous Induction Urgent cesarean Elective cesarean	55 (67.10) 19 (23.20) 6 (7.30) 2 (2.40)	108 (62.10) 54 (31) 5 (2.90) 7 (4)	0.70 ^§^
Labour ending Spontaneous Instrumental Urgent cesarean Elective cesarean	58 (70.70) 9 (11) 13 (15.90) 2 (2.40)	127 (73) 21 (12.10) 19 (10.90) 7 (4)	0.72 ^§^
Birth weight (g)	3165 [756.25]	3372.50 [617.50]	0.001 *
Weight percentile (adjusted by gestational week)	40 [61]	50.50 [49]	0.17 *
Fetal growth retardation (weight percentile < 10)			
Yes	13 (15.90)	12 (6.90)	0.02 ^‡^
Apgar 1st min (<7)			
Yes	6 (7.30)	5 (2.90)	0.10 ^‡^
Apgar 5 th min (<7)			
Yes	4 (4.90)	0 (0.00)	0.002 ^||^

COVID-19 = coronavirus disease 2019; BMI = body mass index; NA = not applicable. Data are median [interquartile range] or *n* (%). * Mann–Whitney U test. † Due to missing data, sums may not add up to total number of patients per column. ‡ Pearson-X2 test. § Fisher´s exact test. || Yates´ correction factor.

**Table 2:** Vitamin D in the study population.

VARIABLE	COVID-19 GROUP (*n* = 82) [Interquartile Range] or *n* (%)	CONTROL GROUP (*n* = 174) [Interquartile Range] or *n* (%)	*p*	OR (95% CI)
25(OH)D (ng/mL)	10.15 [9.45]	13.80 [11.45]	0.005 *	NA
Status of vitamin D deficiency ≥30 ng/mL: Sufficiency 20–29 ng/mL: Insufficiency 10–19 ng/mL: Deficiency <10 ng/mL: Severe deficiency	2 (2.40) 7 (8.50) 34 (41.50) 39 (47.60)	10 (5.70) 33 (19) 75 (43.10) 56 (32.20)	0.004 ^†^	NA
Vitamin D deficiency (<20 ng/mL) Yes	73 (89)	131 (75.30)	0.01 ^‡^	2.66 (1.22–5.77)

COVID-19 = coronavirus disease 2019; OR = odds ratio; CI = confidence interval; NA = not applicable. Data are median [interquartile range] or *n* (%). * Mann–Whitney U test. † Fisher’s exact test. ‡ Pearson-*X*^2^ test.

**Table 3:** Binary Logistic Regression Analysis.

VARIABLE	ORa	95% C.I. OR	*p*
Vitamin D deficiency (<20 ng/mL)	2.68	1.19–6.06	0.01 *
Dependent variable: COVID-19 (yes/no); independent variables: age; vitamin D deficiency (yes/no); studies (yes/no); vitamin D deficiency (yes/no); andrace (Arabian/Caucasian)			

ORa = adjusted odds ratio; CI = confidence interval; OR = odds ratio; COVID-19 = coronavirus disease 2019. * Wald back. Nagelkerke’s R^2^: 0.03. Hosmer–Lemeshow test: Pearson-*X*^2^ test < 0.001, *p*: 1.

**Table 4:** Vitamin D in patients with COVID-19 according to severity.

VARIABLE	MODERATE, SEVERE OR CRITICAL COVID-19 (*n* = 7) [Interquartile Range] or *n* (%)	MILD COVID-19 (*n* = 75) [Interquartile Range] or *n* (%)	*p*
25(OH)D levels (ng/mL)	8.70 [2.90]	10.50 [9.80]	0.25 *
Vitamin D deficiency (<20 ng/mL)			
Yes	7 (100)	66 (88)	0.19 ^†^

COVID-19 = coronavirus disease 2019. Data are median [interquartile range] or *n* (%). * Mann–Whitney U test. † Yates’ correction factor.

**Table 5:** Vitamin D in patients with COVID-19 according to intensive to ICU admission.

VARIABLE	COVID-19 ICU ADMISSION (*n* = 4) [Interquartile Range] or *n* (%)	COVID-19 NOT ICU ADMISSION (*n* = 78) [Interquartile Range] or *n* (%)	*p*
25(OH)D levels (ng/mL)	9.30 [5.73]	10.15 [9.58]	0.41 *
Vitamin D deficiency (<20 ng/mL)			
Yes	4 (100)	69 (88.50)	0.32 ^†^

COVID-19 = coronavirus disease 2019; ICU = intensive care unit. Data are median [interquartile range] or *n* (%). * Mann–Whitney U test. † Yates’ correction factor.

## Data Availability

All data are contained within the article.
